# Study on the extraction method of *Glycyrrhiza uralensis* Fisch. distribution area based on Gaofen-1 remote sensing imagery: a case study of Dengkou county

**DOI:** 10.3389/fpls.2025.1517764

**Published:** 2025-03-07

**Authors:** Xinxin Wei, Zeyuan Zhao, Taiyang Chen, Xiaobo Zhang, Shuying Sun, Minhui Li, Tingting Shi

**Affiliations:** ^1^ School of Life Sciences, Inner Mongolia University, Hohhot, China; ^2^ State Key Laboratory Breeding Base of Dao-di Herbs, National Resource Center for Chinese Materia Medica, China Academy of Chinese Medical Sciences, Beijing, China; ^3^ Inner Mongolia Traditional Chinese & Mongolian Medical Research Institute, Hohhot, China

**Keywords:** *Glycyrrhiza uralensis* Fisch., remote sensing image classification, GaoFen-1, random forest, surface reflectance

## Abstract

*Glycyrrhiza uralensis* Fisch., a perennial medicinal plant with a robust root system, plays a significant role in mitigating land desertification when cultivated extensively. This study investigates Dengkou County, a semi-arid region, as the research area. First, the reflectance differences of feature types, and the importance of bands were evaluated by using the random forest (RF) algorithm. Second, after constructing the *G. uralensis* vegetation index (GUVI), the recognition accuracy of *G. uralensis* was compared between the RF classification model constructed based on the January-December GUVI and common vegetation indices feature set and the support vector machine (SVM) classification model constructed on the GUVI feature set. Finally, the spectral characteristics of *G. uralensis* and other feature types under the 2022 GUVI feature set were analyzed, and the historical distribution of *G. uralensis* was identified and mapped. The results demonstrated that the blue and near-infrared bands are particularly significant for distinguishing *G. uralensis*. Incorporating year-round (January-December) data significantly improved identification accuracy, achieving a producer’s accuracy of 97.26%, an overall accuracy of 93.00%, a Kappa coefficient of 91.38%, and a user’s accuracy of 97.32%. Spectral analysis revealed distinct differences with *G. uralensis* of different years and other feature types. From 2014 to 2022, the distribution of *G. uralensis* expanded from the northeast of Dengkou County to the central and southwestern regions, transitioning from small, scattered patches to larger, concentrated areas. This study highlights the effectiveness of GUVI and RF classification models in identifying *G. uralensis*, demonstrating superior performance compared to models using alternative feature sets or algorithms. However, the generalizability of the RF model based on the GUVI feature set may be limited due to the influence of natural and anthropogenic factors on *G. uralensis*. Therefore, regional adjustments and optimization of model parameters may be necessary. This research provides a valuable reference for employing remote sensing technology to accurately map the current and historical distribution of *G. uralensis* in regions with similar environmental conditions.

## 1 Introduction

Medicinal plants play a pivotal role in traditional medicine and modern medicine. They form the cornerstone of Traditional Chinese Medicine (TCM) and are integral to medicinal systems globally (Filho et al., 2020; Gupta et al., 2024; Huang et al., 2024; Pandey et al., 2024). Due to their natural compounds, medicinal plants exhibit diverse pharmacological properties, such as antibacterial, anti-inflammatory, and analgesic effects, and are widely used in herbal preparations, healthcare products, and cosmetics. Medicinal plants are one of the pillar industries in some countries and an important part of the ecological environment (Gautam et al., 2024; He, 2024). However, their resources are under severe threat due to climate change, overexploitation, and environmental degradation. Thus, research on the conservation and sustainable development of medicinal plants is of paramount importance (Liu et al., 2024; Martins et al., 2024; Mbelebele et al., 2024; Shembo et al., 2024; Sudhakaran, 2024).

Recent advancements in remote sensing technology, diversification of data sources, and algorithmic progress (Li et al., 2023a, 2024b) have significantly improved the accuracy and efficiency of crop image recognition and classification. Accurate crop image recognition and classification are of great value to agricultural monitoring and management (Chen et al., 2024). The Gaofen-1 satellite (GF-1), China’s first high-resolution Earth observation satellite, launched in April 2013, has been instrumental in agricultural production due to its timeliness and capacity for large-area synchronous observations. Its applications include plant resource assessment, growth monitoring, ecological evaluation, and precision agriculture (Akanbi et al., 2024; Kartal et al., 2024; Tilahun et al., 2024). For instance, Liang et al. (2023) employed GF-1 data in Menghai Town, Yunnan Province, to map tea plantation areas using Object-Oriented Image Analysis and Support Vector Machine (SVM) algorithms, observing significant growth from 2014 to 2017. Similarly, Wang and Lei (2021) used GF-1 data to analyze crop spectra in the Manas River Basin, demonstrating the utility of supervised and unsupervised classification methods for cotton area extraction. Xie et al. (2023) explored the county-level agricultural remote sensing mapping capabilities of Gaofen-1 satellite data, indicating that GF-1 satellite data has high remote sensing interpretation accuracy and good applicability in areas with a single crop growing structure. In the area with complex crop planting structure, due to the limitation of image resolution and spectral setting, the mixed pixels increase significantly and the extraction accuracy decreases, but the overall accuracy can reach 90%. It can be seen that GF-1 remote sensing images have been widely used and are highly recognized in the identification and extraction of plant distribution areas due to their excellent technical performance and data processing ability. However, GF-1 data have been less frequently applied to the identification and extraction of medicinal plants, forming the basis of this study.

The choice of classification algorithm significantly impacts results. With the development of science and technology, a variety of classification algorithms have emerged, but the most popular methods are machine learning and DL. In remote sensing image crop classification, they can effectively analyze and process large amounts of image data to achieve high-precision crop identification (Zhang and Dai, 2022; Wang et al., 2024; Zhang et al., 2024c). Xu et al. (2024) used a combination of the feature band and vegetation index combined with machine learning methods to quantitatively analyze the chlorophyll content of *Glycyrrhiza* at different growth stages. Yaolei et al. (2024) explored the effects of different feature types and classification algorithms on the extraction accuracy of macadamia forests showing that a combination scheme of terrain + texture + geometry + topography combined with RF algorithm after feature optimization can effectively identify the distribution of macadamia forests. When compared to traditional machine learning methods, DL has a more powerful feature extraction capability. Zheng et al. (2023) proposed an accurate and real-time coconut tree detection method based on a Faster R-CNN, which successfully detected coconuts from satellite images using a DL method. Bai et al. (2023) showed that the classification accuracy of the improved UPerNet model reached 97.78% on the data set that combines spectral features and vegetation indices. Overall, machine learning and DL play important roles in crop classification using remote sensing images. However, both traditional machine learning and DL have advantages and disadvantages. Traditional machine learning methods are relatively low in data size requirements, and DL models usually require a large amount of data to learn complex patterns. Otherwise, it is difficult to exert its advantages and may lead to overfitting. Second, the DL models can be more affected by problems such as noise, missing values, and inaccurate labeling of data. Traditional methods tend to be relatively more flexible in terms of data quality requirements and have mature preprocessing techniques to work with them that can better cope with data quality issues. Finally, traditional methods have a long history of development and mature theoretical foundations. DL methods, on the other hand, are still developing and evolving, although they have made significant progress in recent years, which means that their stability may be relatively poor (Zhang et al., 2024a). Therefore, it is necessary to reasonably select classification method according to the data characteristics, classification purpose, application scenarios and available resources (Chen, 2024; Rengma and Yadav, 2024; Zhang et al., 2024b; Zhen et al., 2024).

*Glycyrrhiza uralensis* Fisch. comes from Leguminosae. The roots and rhizomes of *G. uralensis* have been used as medicinal herbs for analgesic (Bell et al., 2021; Long et al., 2023; Sha et al., 2024), antitussive (Ren et al., 2024; Shuangshuang et al., 2024), anti-inflammatory (Leite et al., 2022; Kim et al., 2024), and anti - ulcer (Liu et al., 2021) effects. *G. uralensis* is one of the oldest and most widely used herbs in the world (Ding et al., 2022). *G. uralensis* has been used in traditional Chinese medicine for more than 2000 years ago (Shibata, 2000). Approximately 29 species of *Glycyrrhiza* exist globally, of which 15 have medicinal value and are distributed across all continents except Antarctica and in approximately 41 countries (Yan et al., 2023). *G. uralensis* has been widely used for thousands of years in the treatment of a variety of diseases, and is currently used in applied across diverse fields in medicine, food, and cosmetics (Kim et al., 2016; Yang and Zhao, 2022; Chen and Zhang, 2023; Xia et al., 2023; Park et al., 2024). As a perennial plant with a well-developed root system, light-loving, drought tolerance, and strong stress resistance, *G. uralensis* plays an important role in arid and semi-arid regions, such as ecological protection, windbreaks, and sand fixation. It is also a key wild sand-fixing plant that is protected and managed in China (Li, 2017). In recent years, with the in-depth research of *G. uralensis* and a deepening understanding of its medicinal value, the market demand for *G. uralensis* continues to rise. However, this sharp increase in demand has had negative impacts, especially the intensification of predatory harvesting practices, posing a significant risk of *G. uralensis* resource depletion. In addition, due to the comprehensive consideration of local agricultural development by some local governments, as well as economic growth and agriculture modernization, farmers are more willing to choose crops with higher economic benefits, resulting in significant fluctuations in the planting area of *G. uralensis*, which poses a challenge to the sustainable development of the *G. uralensis* industry. The use of modern information technology to accurately identify and monitor *G. uralensis* has become a necessary measure to promote the sustainable development of *G. uralensis* planting and formulate local agricultural development plans (Leite et al., 2022).

In recent years, fluctuations in *G. uralensis* cultivation areas have become more obvious for various reasons, such as the increase in market demand for *G. uralensis*, predatory digging by people, and local agricultural development, which makes it particularly important to use modern information technology to accurately identify and monitor *G. uralensis*. Currently, among the many remote sensing identification methods for crops, methods based on feature indices have the widest range of applications. From existing research reports, there are currently few studies on the spectral feature index for remote sensing identification of *G. uralensis*. According to our investigation in Dengkou County, it is preliminarily judged that there is a large difference between the spectral characteristics of *G. uralensis* and the crops sown simultaneously. Therefore, this difference can be enhanced by constructing a feature index to achieve rapid and accurate identification and extraction of *G. uralensis* planting areas. To achieve high-precision extraction of *G. uralensis* at the county scale and develop a reliable method for monitoring regional crops, this study focused on *G. uralensis* in Dengkou County, a region with a long history of cultivation, extensive planting experience, and a location in a (semi-)arid zone. First, based on the spectral characteristics of the main feature types in Dengkou County under the different bands and months of Gaofen-1 Satellite Wide Field of View (GF-1 WFV) images and band importance analysis, we constructed the *G. uralensis* vegetation index (GUVI). Secondly, the classification accuracies of the *G. uralensis* identification models constructed with GUVI and common vegetation indices were compared to find the optimal *G. uralensis* identification model. Finally, by analyzing the differences in spectral features between *G. uralensis* and other feature types under the optimal feature set for *G. uralensis* identification in 2022, the spatial-temporal evolution characteristics of the *G. uralensis* distribution area in Dengkou County from 2014 to 2021 were extracted and analyzed. The main innovations and contributions of this study are as follows. Based on the spectral characteristics of the main feature types in Dengkou County under different bands and months of the GF-1 WFV image, the differences between the spectral features of *G. uralensis* and other feature types were clarified. Second, the band importance contribution rate was used to understand whether combining January–December data to construct a classification model is more beneficial for identifying *G. uralensis*. Therefore, the remote sensing identification model of *G. uralensis* was constructed by combining January–December GUVI data. Third, by comparing the classification accuracy of the RF classification model constructed based on the January-December GUVI and common vegetation indices feature sets with that of the SVM classification model constructed with the GUVI feature set for *G. uralensis*, we find that the RF classification model constructed using the GUVI feature set for *G. uralensis* distribution area identification is more efficient and robust than the combination of other indices feature sets and classification models. Finally, through the visual interpretation of the spectral feature analysis results of historical images from 2014 to 2021, the distribution area of *G. uralensis* in Dengkou County in the past eight years was successfully identified and extracted, which showed that the model had high versatility and portability, and provided a reference for extracting the historical distribution area of the plant. In addition, this paper provides a reference for identifying and extracting the distribution area of *G. uralensis* in areas with similar conditions to the study area. At the same time, it provides a scientific basis for the rational utilization and ecological protection of *G. uralensis*.

## 2 Materials and methods

### 2.1 Study area

**Figure 1:**
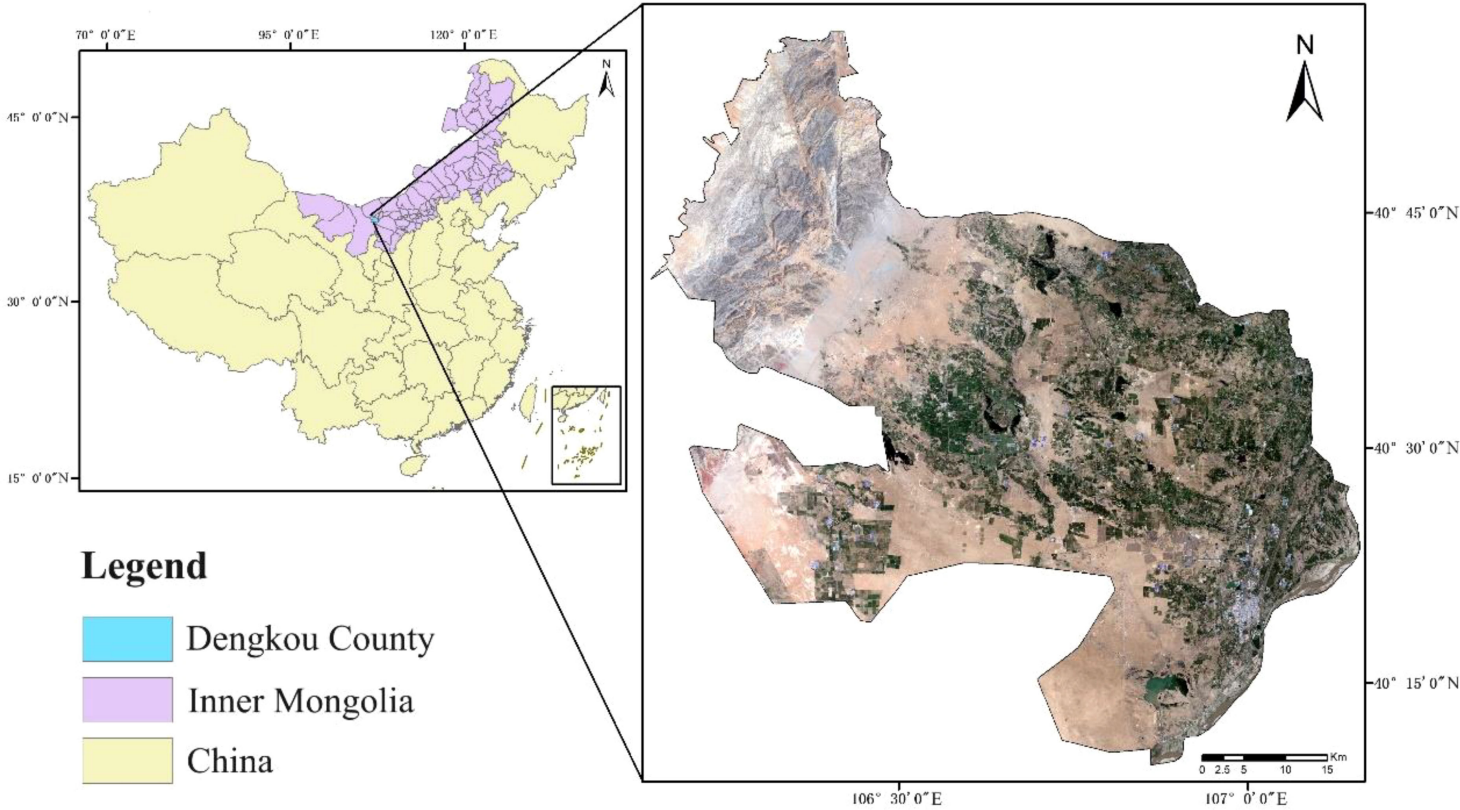
Study area.

Dengkou County, located in Bayannur City, Inner Mongolia Autonomous Region, China, was selected as the study area (**Figure 1**). The highest altitude of Dengkou County is approximately 1951 m, with a total area of approximately 4,200 km^2^. The climate is a temperate continental monsoon type, with an average annual temperature of approximately 9.16°C. The average annual rainfall is 106–155 mm. The temperature difference between day and night is large, approximately 14.5°C, and the frost-free period lasts for 136–144 days. From the remote sensing image shown in **Figure 1**, Dengkou County is located in the upper reaches of the river bend irrigation area and at the eastern edge of the Ulanbuh Desert in the southwestern Inner Mongolia Autonomous Region. Dengkou County is one of the nine experimental demonstration counties for sand control and prevention in China because of its diverse topography, which is dominated by sands, mountains, and plains, and is known as “seven sands, two mountains, and one plain”. The topography and climate of Dengkou County provide a unique foundation for the development of modern agriculture and animal husbandry. In addition to planting traditional crops such as *Avena sativa* L., Helianthus annuus L., *Zea mays* L. and *Glycine max* (L.) Merr., medicinal plants such as *Cistanche deserticola* Ma, *G. uralensis*, and *Astragalus membranaceus* (Fisch.) Bunge is grown in Dengkou County, and the area under cultivation is increasing annually (Jia et al., 2022; Le and Lihua, 2022).

### 2.2 Data sources and pre-processing

The primary data source for this study was the Gaofen-1 Satellite Wide Field of View (GF-1 WFV) images, which can be obtained free of charge from the Land Observation Satellite Data Service (LOSD) of the China Centre for Resources Satellite Data and Application (CRSDA). Application (https://data.cresda.cn/#/home) of the CRSDA. The technical specifications of its payload are shown in **Table 1**. The GF-1 satellite was not only launched earlier, but also has a higher spatial resolution, a larger width and a shorter revisit period (Tong et al., 2023; Yuan et al., 2024). Therefore, more historical image data can be acquired, the details are more clearly shown, more information is covered, and more image data can be acquired within the same period.

**Table 1:** GF-1 WFV satellite payload technical specifications.

Satellite data	Band	Wavelength (mm)	Band Description	Spatial resolution (m)	Span (Km)	Revisit cycle (days)
GF-1 WFV	Band 1	450-520	blue band	16	800	2
	Band 2	520-590	green band			
	Band 3	630-690	red band			
	Band 4	770-890	near infrared (NIR)			

For the acquired GF-1 WFV images, radiometric calibration was first performed using the GF-1 absolute radiometric calibration coefficients released by CRASAC for the corresponding period. Next, the GF-1 WFV spectral response function was imported into the spectral library file, and atmospheric correction was applied using the FLAASH module. Finally, ortho-rectification was conducted with the GMTED2010 elevation data. After radiometric calibration, atmospheric correction, and ortho-rectification, the data significantly reduce atmospheric influence and achieve high geometric accuracy, allowing for better stacking of multi-period images.

### 2.3 Methods

#### 2.3.1 Classification algorithm

RF is an integrated learning algorithm that classifies and predicts samples by training multiple decision trees. Two key parameters play important roles in the RF algorithm: the number of decision trees and the number of split nodes in each tree. By using multiple decision trees to train, classify, and predict data, the RF algorithm can make full use of the differences between different trees, thus improving the accuracy and stability of the classification. In addition, the RF algorithm effectively reduces errors that may be generated by a single classifier using voting multiple classification, which further improves the reliability of the classification. As a novel machine learning algorithm, the RF algorithm has significant advantages in the field of remote sensing classification, as it can integrate numerous decision trees to generate classification results with higher accuracy (Dubrovin et al., 2023; Qian et al., 2023; Pugh et al., 2024).

SVM is a machine learning method based on the Vapnik-Chervonenkis dimension theory of statistical learning theory and the principle of structural risk minimization. SVM can seek the optimal balance between the complexity of the model and the learning ability according to the limited sample information, and it better overcomes the problems of small samples, nonlinearity, over-learning, high dimensionality, and local minima that exist in the traditional classification methods. SVM can obtain better classification accuracy, which is widely used in classification research on remote sensing images, and achieves good results (Pan et al., 2023; Rash et al., 2023; Zhang et al., 2023; Chen, 2024).

#### 2.3.2 Sample collection

**Figure 2:**
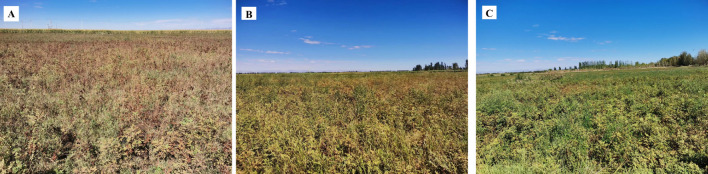
Map of the *G. uralensis* field cultivation areas. **(A–C)** are showing the growth of G. uralensis in different planting areas during the same period.

**Figure 3:**
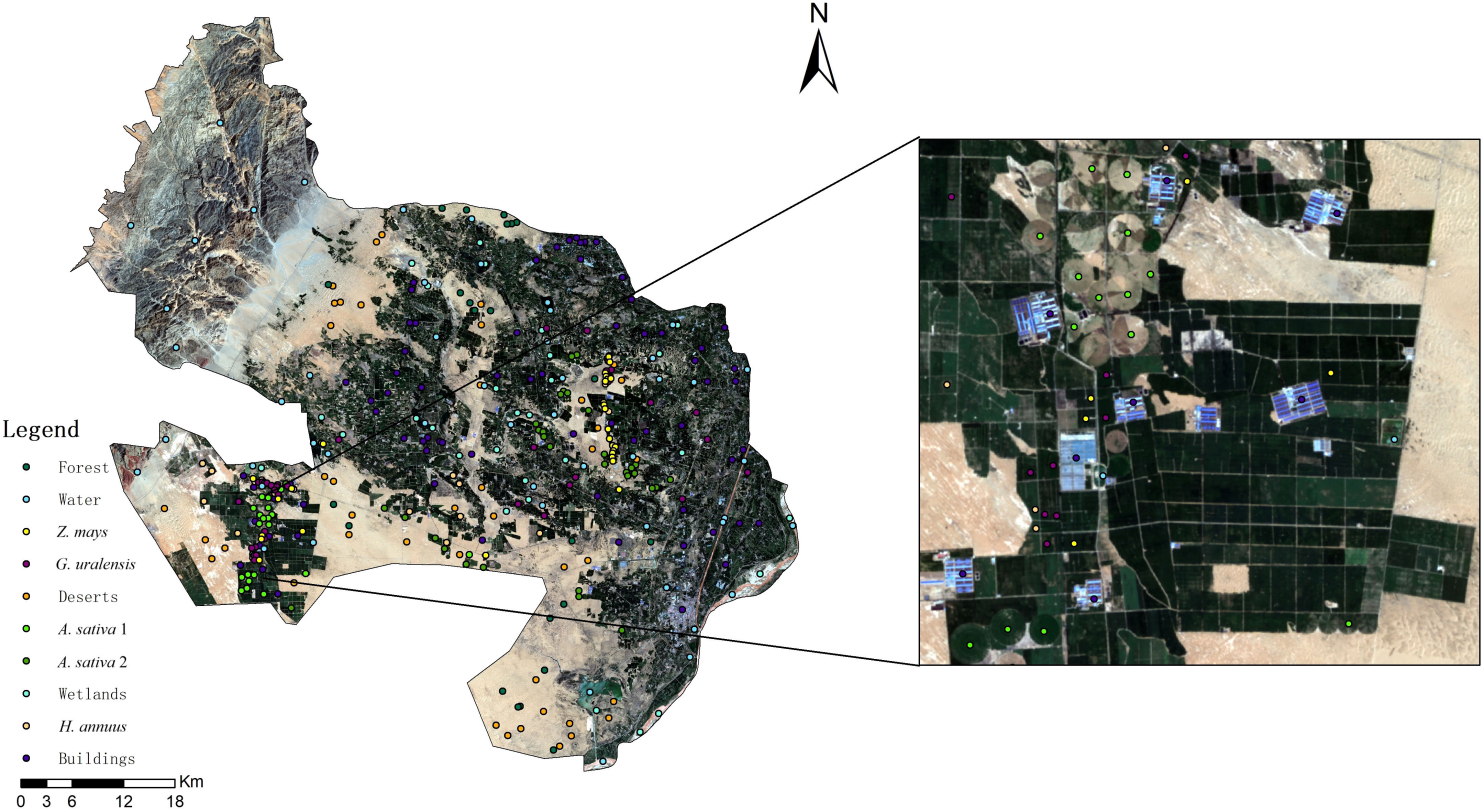
Map of sampling sites in Dengkou County.

The ground reference data for the feature types play a crucial role in the development of training and validation samples to support the image analysis process of feature type mapping. Field observations were used to classify feature types to ensure classification accuracy. We randomly selected sampling points in each plot by contacting a local farmer in advance, which facilitated the establishment of the *G. uralensis* plantation. The minimum distance between any two points was not less than 20 m, and sample points in the same sample area could only be used for training or validation (Zhang et al., 2022; Zhao et al., 2024). Pictures of the *G. uralensis* field planting areas in Dengkou County are shown in **Figure 2**. **Figure 2** illustrates the growth of *G. uralensis* across different planting areas during the same period, ensuring data diversity and enhancing the generalizability and transferability of the research findings. The geographic coordinates of the different feature types were collected using GPS. The data collected for the ground sampling points are shown in **Figure 3**. Based on the number of sampling points, 70% were randomly selected as training data and 30% as validation data.

#### 2.3.3 Validation methods

In this study, four different measurement methods, namely, overall accuracy (OA), Kappa coefficient (KC), *G. uralensis* producer accuracy (PA), and *G. uralensis* user accuracy (UA), of the pixel-based accuracy calculation assessment method were used to judge the classification results comprehensively. All ground object classes in the reference image were considered positive classes, while the classes in the final classification image of the proposed method were predicted classes (Li et al., 2022; 2023b; Rash et al., 2023; Wu et al., 2024; Yilma and Yitay, 2024). A positive class may be predicted as positive (TP) or negative (FN). A negative class can likewise be predicted as being positive (FP) or negative (TN) (Li et al., 2021, 2024a). OA (also known as the percentage of correct classifications) represents the ratio of the number of correctly predicted samples to the number of all predicted samples. KC is a classification consistency test based on the confusion matrix, and its value can represent the level of classification accuracy to a certain extent. PA (also known as precision) represents the ratio of the number of samples correctly predicted as positive to the total number of samples predicted as positive. UA (also known as recall) represents the ratio of the number of samples correctly predicted as positive to the number of all positive samples (Gun and Chen, 2023). Usually, higher values of OA, KC, PA, and UA indicate higher classification accuracies. Shown here are **Equations (1)** to **(5)**.


(1) $$\begin{array}{c} OA=\frac{TP+TN}{TP+FN+FP+TN} \end{array}$$

(2) $$\begin{array}{c} KC=\frac{OA-PRE}{1-PRE} \end{array}$$

(3) $$\begin{array}{c} PRE=\frac{\left(TP+FP\right)\left(TP+FN\right)+\left(FN+TN\right)\left(FP+TN\right)}{{\left(TP+TN+FP+FN\right)}^{2}} \end{array}$$

(4) $$\begin{array}{c} UA=\frac{TP}{TP+FN} \end{array}$$

(5) $$\begin{array}{c} PA=\frac{TP}{TP+FP} \end{array}$$

## 3 Results of *G. uralensis* vegetation index construction and *G. uralensis* distribution area extraction

### 3.1 Spectral characterization of feature types

**Figure 4:**
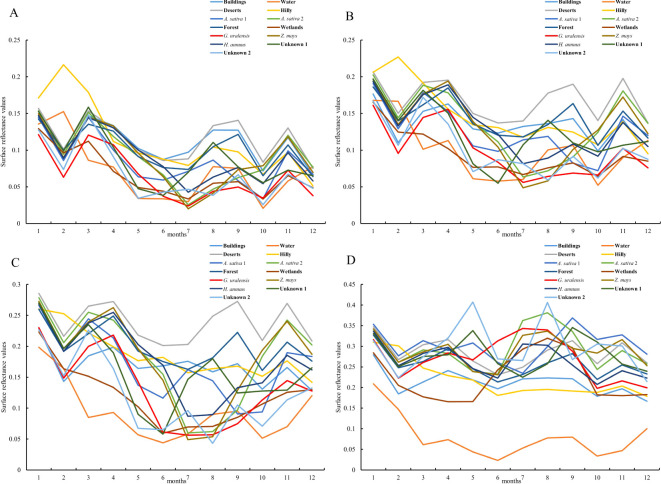
Surface reflectance of feature types in Dengkou County under GF-1 WFV imaging band. **(A–D)** are the surface reflectances of the major feature types in Dengkou County in the blue, green, red, and near-infrared bands, respectively.

After ensuring the authenticity and the accuracy of the surface reflectance data by preprocessing the remote sensing images and eliminating the noise, distortion, and interference factors in the images, the surface reflectance values of the main feature types in Dengkou County in 2022 were extracted using field survey data and remote sensing image data, and the results are shown in **Figure 4**.

From **Figure 4A**, the January–December surface reflectance of each feature type in the blue band was similar, and except for water bodies and mountains, the curves of all of the other feature types were consistent with each other, showing a decreasing trend in spring (March-May), increasing in summer (June–August), decreasing and then increasing in autumn (September–November), and increasing and then decreasing in winter (December–January). In autumn (September–November), the curves first decreased and then increased, whereas in winter (December–January), they first increased and then decreased. The *G. uralensis* reflectance curves distinctly differed from the other feature types in January, February, September, and October. As shown in **Figure 4B**, the surface reflectance for each feature type under the green band exhibits distinct curve characteristics in spring, summer, and autumn, except for the overall trend of increasing and then decreasing in winter. Likewise, the *G. uralensis* reflectance curves were distinguished from other feature types in January, February, September, and October. In **Figure 4C**, the surface reflectance curves for each feature type under the red band are similar to those under the green band and show an overall increasing and then decreasing trend in winter. The *G. uralensis* reflectance curves were distinguished from the other feature types in September. From **Figure 4D**, all of the feature types under the Near Infrared (NIR) band showed the same pattern of increasing and then decreasing, except in winter, with peaks of different sizes from June to October, which is similar to the reflectance curves of all feature types under the blue band. The *G. uralensis* reflectance curves differed significantly from those of the other feature types in June. Therefore, calculating the difference between the blue and near-infrared bands can enhance the distinction between spectral curves, facilitating the identification and extraction of *G. uralensis* and other feature types.

**Figure 5:**
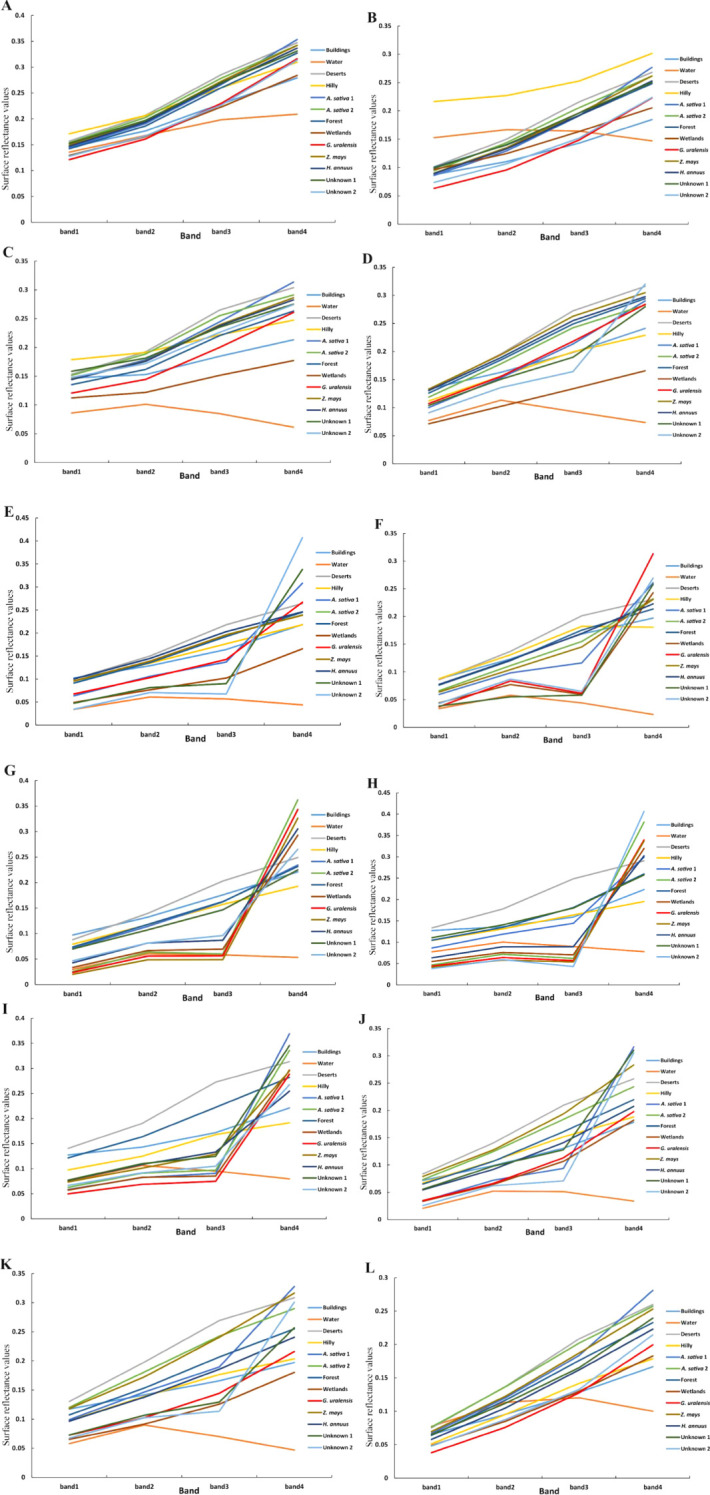
Surface reflectance of major feature types in Dengkou County under GF-1 WFV imagery from January to December. **(A–L)** is the surface reflectance of the major feature types in Dengkou County under GF-1 WFV imagery from January to December.

As shown in **Figure 5**, certain feature types can be accurately recognized under month-specific imagery, and a collation of such feature types and the months in which they are recognized are as follows: buildings (September), water bodies (January–December), deserts (August–September), mountains (February, September), oats 1 (September), wetlands (March-May), and unknown 2 (April–May). In addition, the feature types exhibit similar curve changes for the same remote-sensing image. Except for the imagery from June and August, the reflectance values of each band in the remaining images show a trend of blue, green, red, and near-infrared bands from small to large. Therefore, it is difficult to accurately identify *G. uralensis* and other features using remote sensing images at certain times. Moreover, according to the reflectance values of the four bands for the main feature types, a result consistent with the analysis in **Figure 4** can be obtained; that is, the calculation between the blue and near-infrared bands is more conducive to increasing the differences between *G. uralensis* and the feature types, thus facilitating the identification of each feature type.

**Figure 6:**
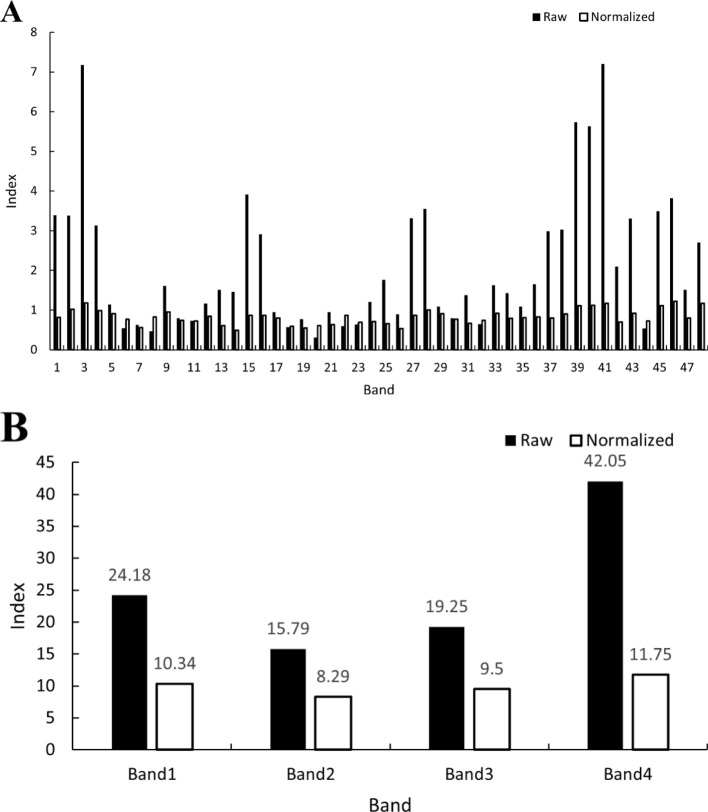
Band raw and normalized significance contributions. **(A)** shows the original and normalized importance contribution rate of a single band, where 1–12 are the Band 1 of the January–December images, 13–24 are the Band 2 of the January–December images, 25–36 are the Band 3 of the January–December images, and 37–48 are the Band 4 of the January–December images. **(B)** shows the total raw and normalized importance contributions of each band from January to December; the two values of Band 1–Band 4 are the sum of the raw and normalized importance contribution of Band 1–Band 4 of the January–December images, respectively.

Based on these results, the band importance was evaluated using RF, and the results are shown in **Figure 6**. **Figure 6A** shows that the raw importance contribution rate is highest for the NIR band, followed by the blue band with a higher contribution rate, and the red and green bands with the lowest raw importance contribution rate. The normalized importance contribution rate was highest for the blue band, followed by the near-infrared band, and lowest for the red and green bands. After further organizing the band contribution rates by band types, **Figure 6B** shows that the raw contribution rates ranked from largest to smallest as NIR, blue, red, and green bands. The normalized importance contribution rates from the largest to smallest were the blue, near-infrared, red, and green bands. This result is consistent with the aforementioned results and shows that combining the January–December bands into a model enhances the classification of each object type.

### 3.2 Vegetation index construction for *G. uralensis*

Based on the result that the calculation between the blue and near-infrared bands is more conducive to increasing the difference between *G. uralensis* and other feature types obtained from the analysis of spectral characteristics of feature types, the difference between the near-infrared band and blue band was increased using the formula (A−B)/(A + B) in combination with commonly used vegetation indices formulas (Zhai et al., 2023; Pei et al., 2024; Tang et al., 2024; Zhen et al., 2024). It was named GUVI, and the formula is shown in **Equation (6)**.


(6) $$\begin{array}{c} GUVI=\frac{\left(NIR-B\right)}{\left(NIR+B\right)} \end{array}$$

where NIR represents the near-infrared band, and B denotes the blue band.

To verify the reliability of the GUVI for identifying the distribution area of *G. uralensis*, seven commonly used vegetation indices (**Table 2**) were selected to construct the *G. uralensis* identification model for comparison and verification (Giovos et al., 2021; Zeng et al., 2022; Han et al., 2024). Because it is difficult to accurately identify and extract the distribution area of *G. uralensis* and other feature types under a single remote sensing image, and according to the results of the band importance contribution rate, this study, the monthly GUVI, and seven other vegetation indices were calculated separately after processing the remote sensing images from January to December 2022 and were combined into eight long time series data as feature sets in the order of months. Based on the sampling point data of the main feature types in 2022, 70% were selected as the training set and the remaining 30% as the validation set. Eight identification models of *G. uralensis* were constructed using the RF algorithm, and the reliability of the GUVI was verified by comparing the classification accuracies of the RF algorithm for the eight models. The classification effect of different classification algorithms on the GUVI feature set construction was compared by comparing the classification accuracy of the RF classification model constructed with the GUVI feature set and the SVM classification model (Ko et al., 2024; Yang et al., 2024). The results are shown in **Table 3**.

**Table 2:** Vegetation indices information.

Vegetation index in English	Abbreviations	Formulas	Reference source
Normalized Difference Vegetation Index	NDVI	(NIR-R)/(NIR+R)	Huang et al. (2021)
Difference Vegetation Index	DVI	NIR-R	Ragini et al. (2024)
Enhanced Vegetation Index	EVI	2.5*(NIR-R)/(NIR+6*R-7.5*B+1)	Ambadkar et al. (2024)
Green Normalized Difference Vegetation Index	GNDVI	(NIR-G)/(NIR+G)	Daliman et al. (2024)
Green Chlorophyll Vegetation Index	GCVI	(NIR/G)-1	Shuai and Basso (2022)
Ratio Vegetation Index	RVI	NIR/R	Chowdhury et al. (2024)
Triangular Vegetation Index	TVI	60*(NIR-G)-100*(R-G)	Sun et al. (2023)

**Table 3:** Classification accuracy of vegetation indices feature set.

Model	OA (%)	KC (%)	*G. uralensis* UA (%)	*G. uralensis* PA (%)
NDVI	92.47	90.64	93.53	97.67
DVI	93.79	92.29	93.68	97.22
EVI	92.15	90.30	96.57	97.73
GNDVI	92.10	90.23	97.13	98.04
GCVI	92.12	90.25	96.70	98.04
RVI	92.45	90.62	93.21	97.79
TVI	92.43	90.60	93.63	97.41
GUVI_RF_	93.00	91.38	97.26	97.32
GUVI_SVM_	85.63	82.42	93.36	79.93

**Figure 7:**
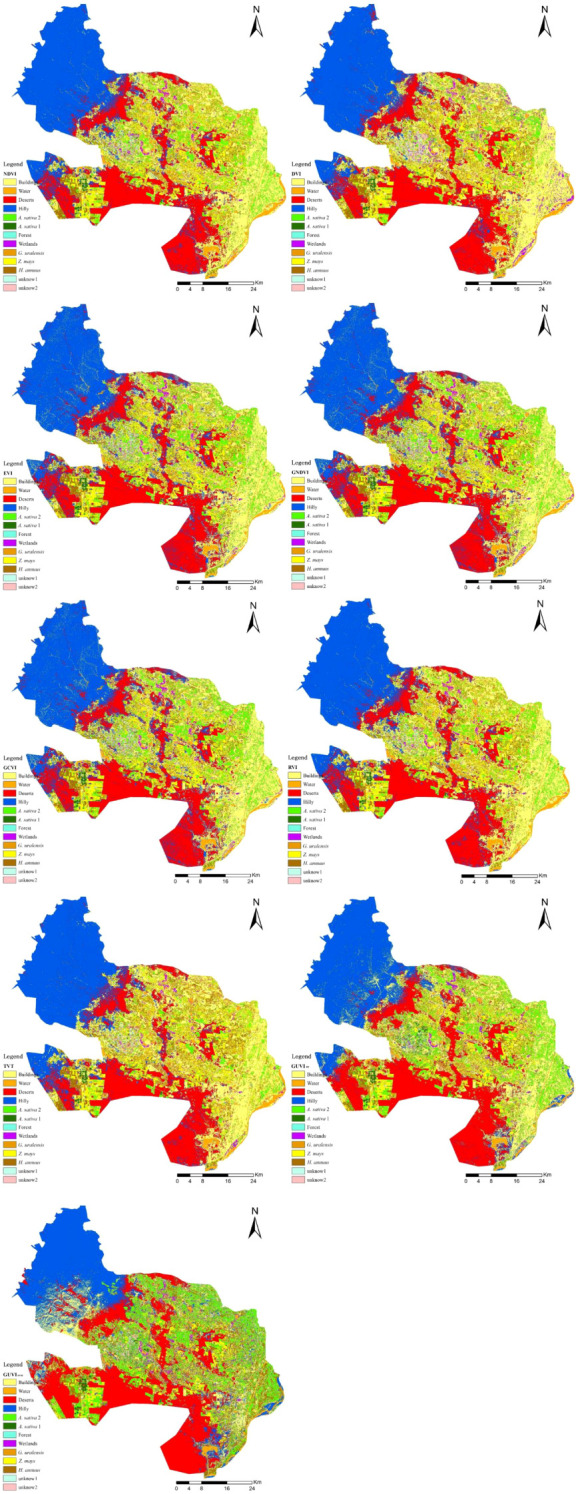
Vegetation indices feature set model classification results map.

**Table 3** shows that the model constructed with DVI as a feature had the highest OA and KC of 93.79% and 92.29%, respectively, and its *G. uralensis* UA and PA were 93.68% and 97.22%, respectively. The model constructed with GNDVI as a feature had the highest *G. uralensis* UA of 97.13%, OA of 92.10%, KC of 90.23%, and *G. uralensis* PA of 98.02%. The model constructed using GUVI as a feature had the highest *G. uralensis* PA of 97.26%, OA of 93%, KC of 91.38%, and *G. uralensis* UA of 97.32%. The model constructed with GUVI as a feature was better than the DVI in terms of *G. uralensis* UA and PA values; it was better than the GNDVI in terms of OA, KC, and *G. uralensis* PA values; OA and KC were not the highest, but the performance was excellent. In addition, through the classification accuracy of the two classification algorithms, SVM and RF, for the GUVI feature set that RF has a better classification effect, which is higher than the classification accuracy of SVM in the values of OA, KC, *G. uralensis* UA and *G. uralensis* PA. From the vegetation indices feature set model classification results graph (**Figure 7**), the GUVI_RF_ classification results were more accurate than the other results and were able to identify the subtle differences between the habitat types more accurately, producing fewer misclassifications. Even using different classification algorithms to classify the GUVI feature set yielded better results, indicating that the method is more robust and efficient in recognizing *G. uralensis*. That is, the classification of the GUVI model with RF can effectively recognize the distribution area of *G. uralensis* in Dengkou County, and it has certain applicability in other *G. uralensis* planting areas.

### 3.3 Difference analysis of spectral characteristics of *G. uralensis* with different growth years

According to the results discussed in Section 3.2, the model of the GUVI combination for January–December 2022 can be used as a feature set to effectively identify and classify *G. uralensis* and other feature types using the RF algorithm. Therefore, this method can identify and classify *G. uralensis* and other feature types in historical remote sensing images from 2014 to 2021. However, owing to the lack of actual sampling point data for past feature types, the corresponding training and validation sets could not be selected. Therefore, in this study, the spectral curves of the main feature types and different growth years of *G. uralensis* were obtained based on the average values of the training and validation sets under the GUVI combination model for January–December 2022 to analyze the differences between the spectral curves of *G. uralensis* and other feature types, while simultaneously understanding the difference in the spectral curve of *G. uralensis* with different growth years. This enabled more comprehensive information on *G. uralensis* to be captured while distinguishing *G. uralensis* from other feature types without actual sampling points, allowing the RF algorithm to identify historical *G. uralensis* distribution areas more accurately.

**Figure 8:**
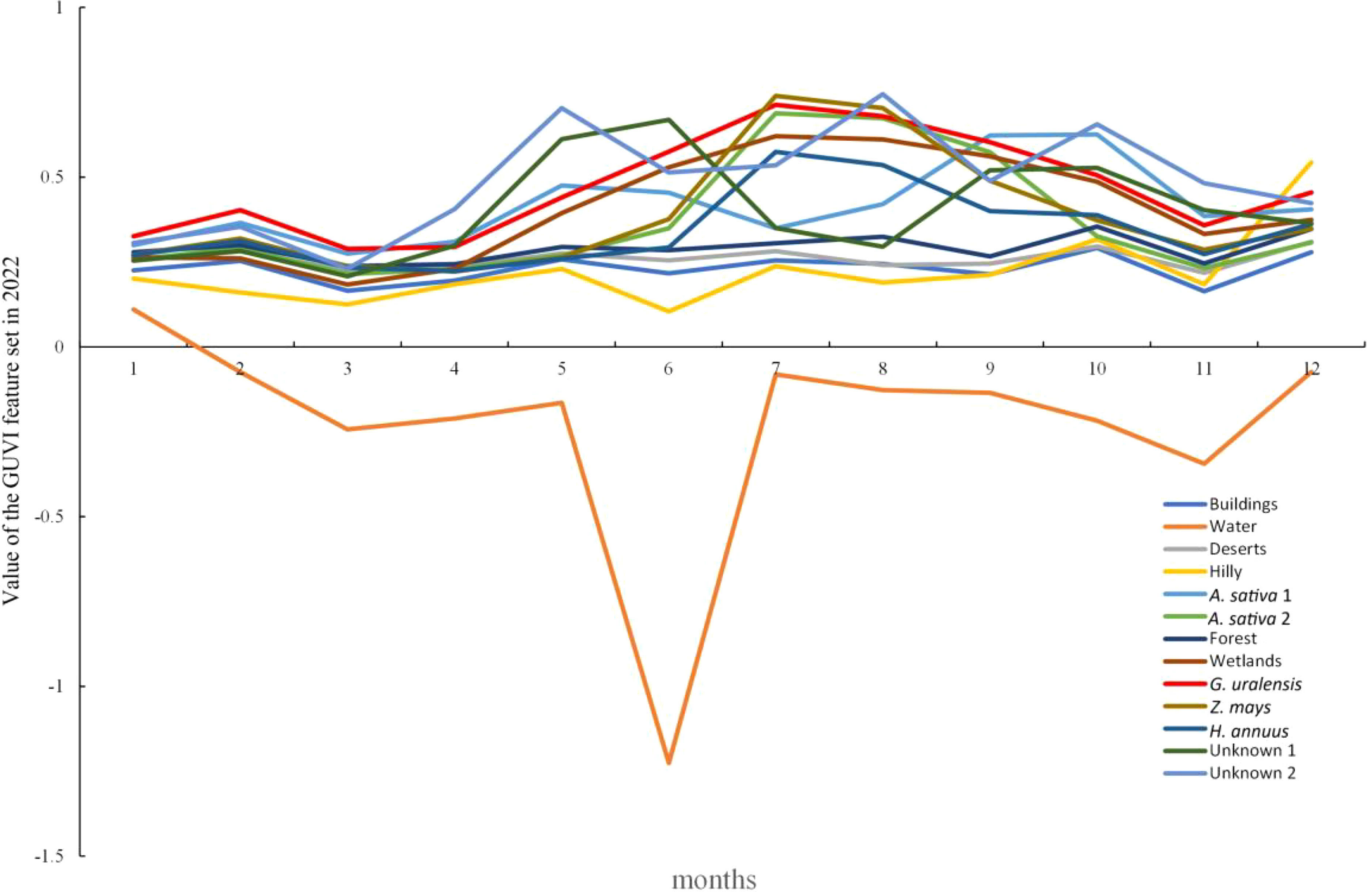
Spectral curve of major feature types under the GUVI model for 2022.

The spectral curves of the main feature types under the GUVI model for 2022 are shown in **Figure 8**, with different GUVI values and spectral curves for each feature type. In terms of the GUVI values, the *G. uralensis* spectral curve was significantly different from the other feature types for eight months (January, February, May, June, July, September, November, and December). The feature types with GUVI values similar to those of *G. uralensis* in the remaining months were oats in March, unknown 1 in January and April, oats 2 and maize in August, and wetlands in October. These feature types in other months are significantly different from those of *G. uralensis* and can be distinguished based on the values for other months. Based on the curve analysis, the feature type that has similar characteristics to the *G. uralensis* curve is wetland, and the curves are all characterized by a rise in spring, a peak in July, a low peak in October, and a trough in winter in January of the following year. However, both had similar values, except in October, and the values of *G. uralensis* were greater than those of the wetland in the remaining months. Therefore, according to **Figure 8**, it can be concluded that the spectral curve characteristics of *G. uralensis* under the GUVI model are different from those of other feature types. Thus, the training and validation sets can be selected under the GUVI model based on the curve characteristics, and *G. uralensis* can be identified by the RF algorithm from 2014 to 2021.

*G. uralensis* is a perennial herb or half-shrub, and its growing status has been shown to vary significantly from year to year (Xi, 2020), and the reflected spectral features will be different. In order to more accurately identify and extract the distribution areas of *G. uralensis* in historical remote sensing images, this study further compiled the characteristics of the spectral curves of *G. uralensis* in different years under the GUVI model for 2022, based on the *G. uralensis* year information provided by local growers at the time of sample collection.

**Figure 9:**
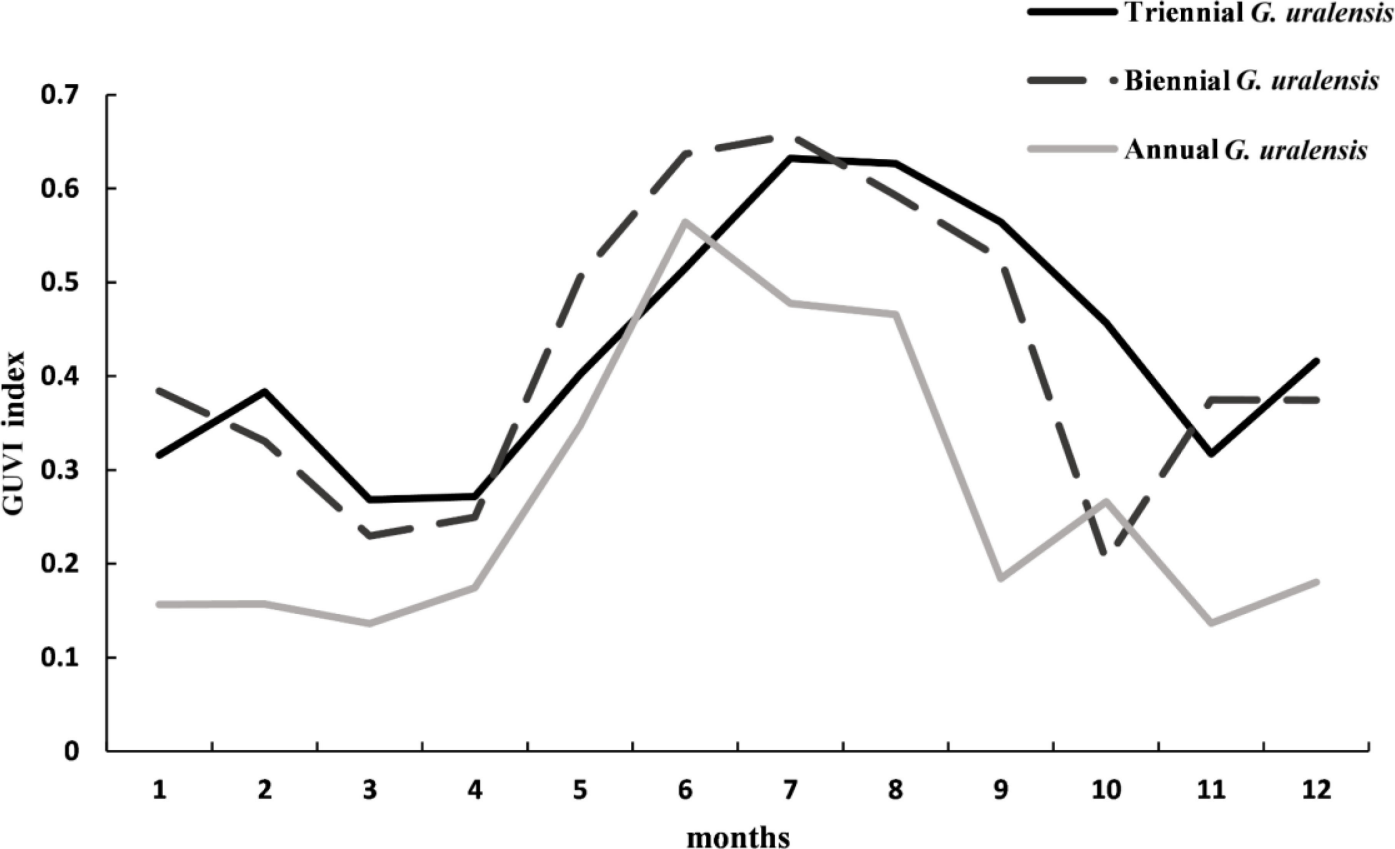
Spectral curves of *G. uralensis* of different ages under the GUVI model in 2022.

As shown in **Figure 9**, the characteristics of the spectral curves for different years of *G. uralensis* were different. In terms of values, annual *G. uralensis* had lower values than biennial and triennial *G. uralensis* in all months except June and October. Biennial *G. uralensis* had higher values than triennial *G. uralensis* in January, May, June, July, and November, while triennial *G. uralensis* had higher values than biennial *G. uralensis* in the remaining months. The spectral curve of annual *G. uralensis* started to rise in March, surged in April–June, peaked in June, and then started to decline, and was more moderate in July–August, declined in September, and then started to rise in October, declined again in November, and rose again in December. The spectral curve of biennial *G. uralensis*, however, started to fall in January, began to rise after March, peaked in July, and then fell to its lowest value in October, after which it started to rise and became relatively flat in November and December. The triennial *G. uralensis* curve rose in January, declined throughout February, was relatively flat in March and April, rose again, reached a high value in July, remained relatively flat in August, and then declined until it began to rise again after November. Therefore, the spectral curve characteristics of *G. uralensis* across different growth years should be comprehensively considered when selecting sample points from historical remote sensing images to enhance the RF algorithm’s accuracy in identifying *G. uralensis* distribution areas.

### 3.4 Extraction of *G. uralensis* historical distribution area in Dengkou County

According to the results in Section 3.3, under the GUVI model, the spectral curves of different feature types and different growth years of *G. uralensis* have their characteristics, which can be used to select samples of the main feature types in Dengkou County from historical images. Therefore, after preprocessing the remote sensing images from 2014 to 2021, the monthly image GUVI was calculated, and the different years GUVI were combined into eight feature sets of the RF algorithm in the order of January–December. The features were deciphered according to the spectral curves of *G. uralensis* with different growth years as well as other feature types. The visual interpretation was used to obtain the sample points of *G. uralensis* and the other feature types in the historical images from 2014 to 2021. After selecting the training set and validation set in the ratio of 7:3, the distribution area of *G. uralensis* was identified using the RF algorithm, and its accuracy was verified.

The classification accuracies of *G. uralensis* distribution areas of RF classification model constructed with GUVI feature set for 2014–2021 are shown in **Table 4**. Among them, the lowest value of OA was 89.72%, the lowest value of the KC lineage was 87.16%, the lowest value of *G. uralensis* UA was 69.47%, and the lowest value of *G. uralensis* PA was 71.08%. Therefore, the overall classification accuracy and individual classification accuracy of *G. uralensis* were good for 2014–2022 and could be used for *G. uralensis* range extraction.

**Table 4:** Classification accuracy of RF classification model constructed with GUVI feature set.

Years	OA (%)	KC (%)	*G. uralensis* UA (%)	*G. uralensis* PA (%)
2014	96.83	95.78	100.00	75.00
2015	95.80	94.73	100.00	100.00
2016	97.97	97.08	96.72	77.63
2017	95.44.	94.03	97.25	92.19
2018	93.34	90.92	88.41	95.87
2019	89.91	87.95	74.31	91.85
2020	89.72	87.16	89.22	99.27
2021	90.72	89.21	69.47	71.08

**Figure 10:**
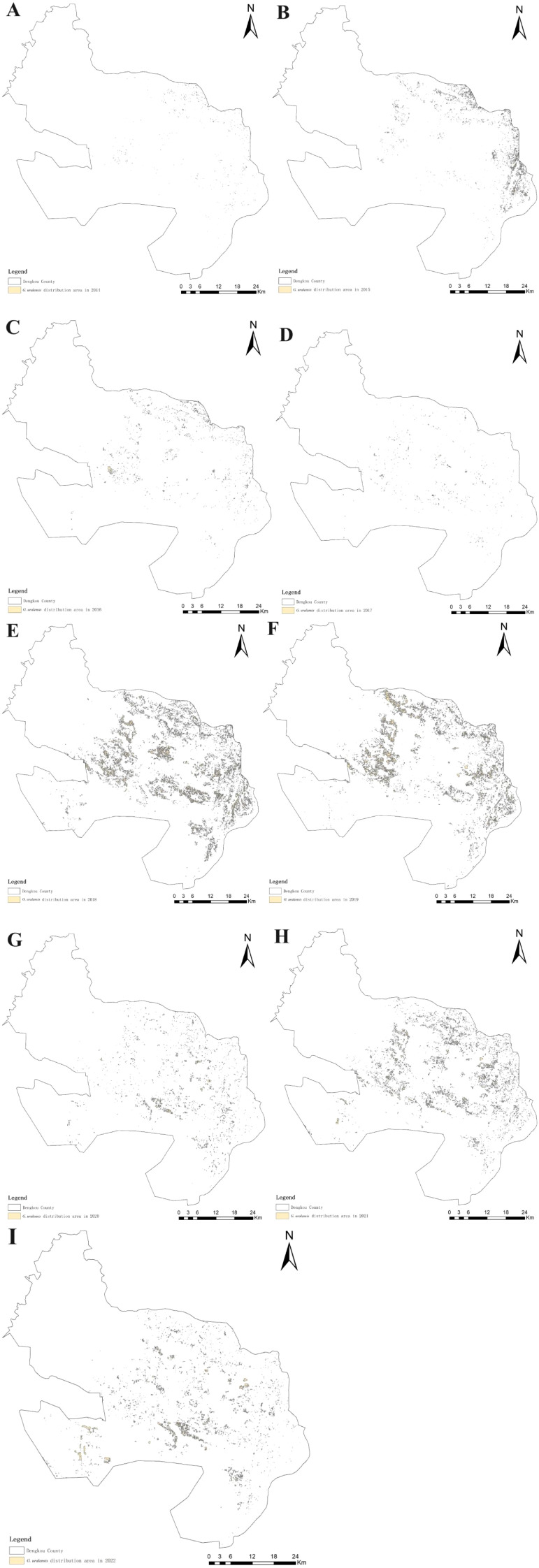
*G. uralensis* distribution area in Dengkou County, 2014–2022. **(A–I)** are the distribution maps of *G. uralensis* in Dengkou County from 2014 to 202.

**Figure 11:**
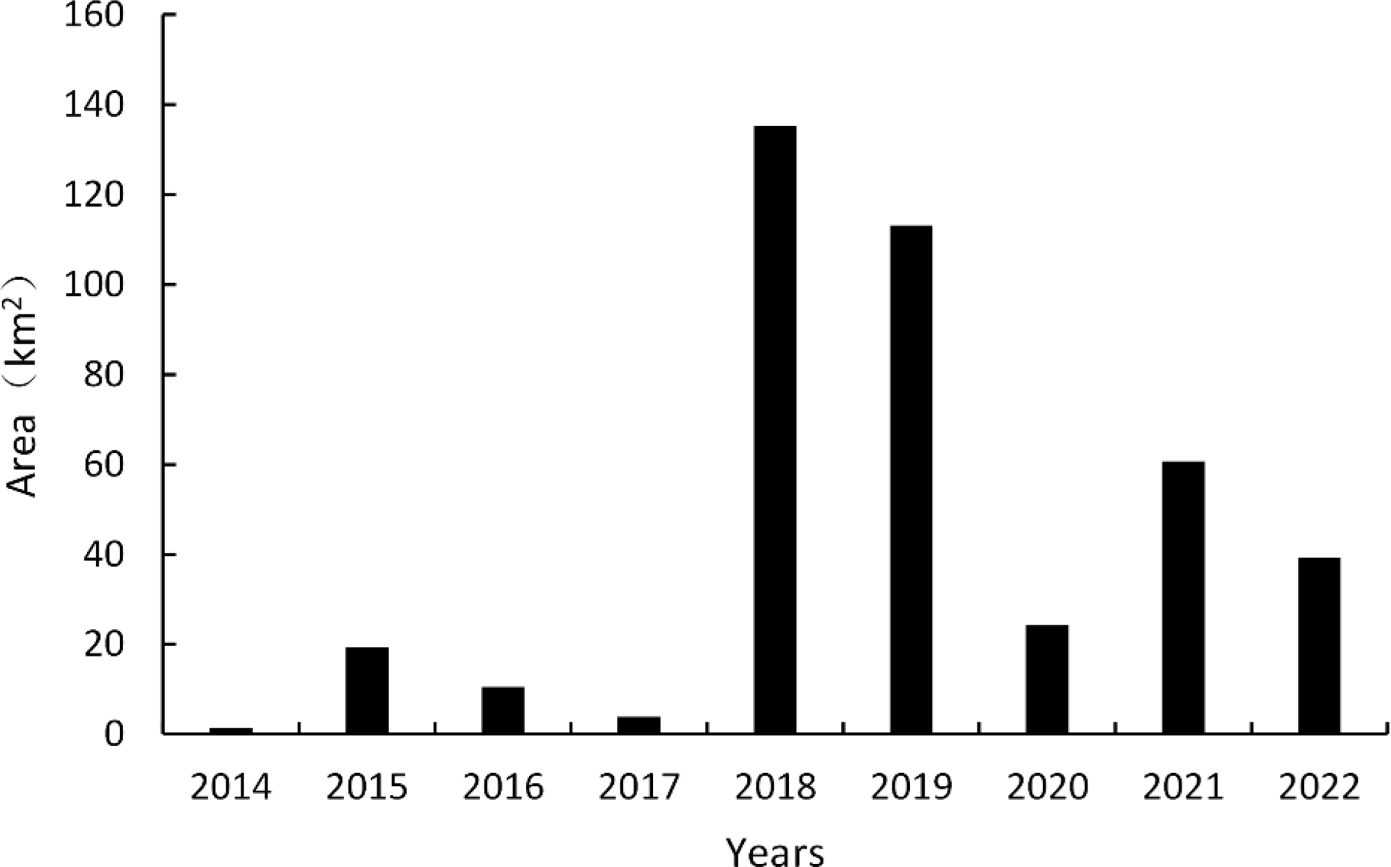
Changes in *G. uralensis* acreage in Dengkou County, 2014–2022.

The spatial distribution and changes in the *G. uralensis* distribution area from 2014 to 2022, extracted from the identification results of the *G. uralensis* distribution area using the RF algorithm under the GUVI model, are shown in **Figures 10**, **11**. As shown in **Figure 10**, the distribution of *G. uralensis* gradually expanded to the central region from its initial main concentration in the northeast of Dengkou County and is now distributed in the southwest. The distribution area gradually changed from small and scattered at the beginning to a large area of concentrated distribution. **Figure 11** shows the changing trend of the *G. uralensis* area, which first increased and then decreased. The most significant increase in the *G. uralensis* area was observed in 2018. As *G. uralensis* is a perennial plant, it is difficult to systematically count the area of *G. uralensis* planting and harvesting each year; however, it generally shows a stable planting pattern. In addition, although Dengkou County has a long history of *G. uralensis* planting, the planting area was small before 2018, and only after 2018 did it begin planting on a large scale.

## 4 Discussion

### 4.1 Band characterization and construction of a vegetation index for *G. uralensis*

Although multispectral remote sensing images offer advantages such as wide coverage and high spatial-temporal resolution, their limited spectral bands can pose challenges for accurate classification. When relying on original image spectra to classify and identify feature types, often leads to suboptimal accuracy. In addition, the fragmentation of agricultural land systems in terms of cultivation type and area, as well as the influence of the phenomenon of “different spectra for the same object and the same spectra for the same object” and the mixed pixel effect, making remote sensing identification and extraction of crops more complicated than that of the natural vegetation (such as woodland and grassland). Zheng et al. (2022) investigated remote sensing identification of film-covered farmland in the Loess Plateau using various combinations of spectral, exponential, and texture features. They found that combining spectral, exponential, and optimal texture features yielded the best classification results. Liang et al. (2021) successfully constructed remote sensing features for citrus by combining spectral, climatic, textural, and spectral indices and reported that the normalized vegetation index (NDVI) was particularly outstanding in the identification of citrus. Liu et al. (2023) used Google Earth Engine, Sentinel - 2 data and winter wheat phenology as prior knowledge to develop new indices, including the Normalized Differential Phenology Index (NDPI), Wheat Phenology Differential Index (WPDI), Normalized Differential Wheat Phenology Index (NDWPI), and Plastic Mulched Index (PMI). This method is used to obtain the spatial distribution and planting area information of winter wheat. The accuracy of the proposed method is higher in the early wintering stage and the regreening stage, which are 82.64% and 88.76%, respectively, and has strong spatial and temporal transfer. In order to improve the distribution area of a certain plant, it is necessary to find the differences with other plants by understanding the growth characteristics, spectral characteristics, temporal phase changes, and other aspects of information, and then increase the differences through the method of band operation, and further increase the differences by combining the features to construct a more accurate and effective remote sensing identification model. This would improve the identification accuracy and classification effect of the distribution area of the plant. In the classification algorithms, although the principles of the different algorithms are different, the classification accuracy of the two different algorithms applied in this study for the GUVI feature set shows that this classification model has a certain degree of robustness and the efficiency of recognizing *G. uralensis* is high, thus indicating that the RF classification model constructed based on the GUVI feature set is not overly influenced by the characteristics of a single algorithm and can classify *G. uralensis* stably in the framework of different algorithms. In addition, the classification accuracy of *G. uralensis* distribution areas under the RF classification model based on the GUVI feature set across different growth years shows that the model can maintain relative stability in the process of year change, indicating that the RF classification model based on the GUVI feature set has the common characteristics of *G. uralensis* distribution areas at different times and is not subject to the excessive interference of factors such as environmental changes between years; therefore, it has good portability. Given that the sampling points in this study covered a variety of *G. uralensis* growth conditions ranging from good to poor growth (**Figure 2**), which ensured the diversity and representativeness of the data, the RF classification model constructed based on the GUVI feature set was considered to have good generalizability. However, due to the influence of many natural and human factors such as region, climate, soil, farmers’ planting methods and cycles, the universality of the RF classification model based on *G. uralensis* feature set in different regions may be limited, and it is necessary to adjust the parameters and optimize the model for specific regions. In practical applications, it is necessary to fully consider its limitations and combine other methods to improve the accuracy and efficiency of classification.

### 4.2 Difference analysis of spectral characteristics of *G. uralensis* with different growth years

There are obvious differences in the spectral characteristics of different plants due to the differences in leaf tissue structure and pigments. For the same plant, the spectral characteristics may change significantly at different growth stages or under different environmental conditions. As *G. uralensis* is a perennial herb or half-shrub, understanding its spectral characteristics at different ages forms the foundation for classifying feature types over an extended time series. The current study demonstrated that the spectral curve characteristics of different annual *G. uralensis* varieties were significantly different. This may be because one-year-old *G. uralensis* is in the early stage of growth, its leaves are not yet mature, and the content of chlorophyll and other pigments is relatively low. Meanwhile, its biomass is small, the leaves are thin, and the organizational structure is not close enough, which makes its absorption and reflection of light weaker, thus exhibiting lower characteristics in the spectrum. In contrast, the spectral curves of biennial and triennial sweetgrasses were more similar because of the maturity of the leaves, increased content of chlorophyll and other pigments, accumulation of more biomass, and other reasons. However, there may be a few differences owing to differences in accumulation, environmental factors, or the management styles of farmers, which need to be further analyzed. From the overall analysis of these three curves, reveals a clear peak from May to August, a trough from March to April and September to November. This trend closely correlates to the growth cycle of *G. uralensis*. From March to April, *G. uralensis* recovers from dormancy, the leaves are not yet fully expanded, and the chlorophyll content is low; therefore, the spectral characteristics are not obvious and show a trough. From June to August, this is the most vigorous growth period of *G. uralensis*, with high chlorophyll content, lush leaves, significant reflection and absorption spectrum characteristics, forming a peak. From September to November, *G. uralensis* enters the late stage of growth, leaves begin to senesce, chlorophyll gradually decomposes, and the spectral characteristics are weakened, again forming a trough. From December to February, the ground surface is covered with snow, which has high reflectivity, making the spectral features appear higher than from March to April and September to November. This phenomenon further enriches our understanding of the variation in the spectral characteristics of *G. uralensis* and provides a useful reference for the subsequent classification of feature types.

### 4.3 Changes in the distribution area of *G. uralensis* in Dengkou County

The distribution area of *G. uralensis* in Dengkou County has experienced significant changes from 2014 to 2022. Studies during this period showed that the distribution area of *G. uralensis* not only expanded, but also its concentrated distribution became more prominent. Initially, *G. uralensis* was mainly distributed in a small area in the northeastern part of Dengkou County, but over time, it gradually expanded to the central and southwestern parts of the county, eventually forming a large area of concentrated distribution. This change reflects the importance of *G. uralensis* in the region and shows the complex interaction between natural conditions and human factors. First, the geographical conditions in the northeastern part of Dengkou County provide a favorable environment for *G. uralensis* growth. This region benefits from the Yellow River and has abundant water resources, creating an environment suitable for *G. uralensis* growth. The abundance of water resources enables *G. uralensis* to obtain the water required during the growing season, thereby improving its growth rate and quality. In addition to the influence of natural conditions, the local agricultural technology extension departments play a key role. With the spread and popularization of *G. uralensis* cultivation techniques, farmers’ cultivation and management skills have significantly improved. These departments may help farmers master modern cultivation methods and management techniques by providing training and guidance that have improved the overall efficiency and yield of *G. uralensis* cultivation. In addition, with the large-scale development of the *G. uralensis* cultivation sector, cooperation and exchanges among growers have become increasingly frequent. Growers have further improved the efficiency and competitiveness of *G. uralensis* cultivation by sharing cultivation experiences and optimizing cultivation patterns. Such cooperation enhances ties between growers and provides a good basis for the intensive production of *G. uralensis* and promotes its distribution on a wider scale. However, the areas planted with *G. uralensis* fluctuated significantly between 2014 and 2022. This fluctuation may be due to the combined effect of many factors. For example, inconsistencies in growers’ harvest years may lead to changes in the areas under *G. uralensis* cultivation. Due to the fluctuations in market demand, growers may adjust the planting area of *G. uralensis* according to the change in market price and demand in each growing season. In addition, the intensification of market competition may impact the decision-making of growers. A few growers may choose to reduce the area of *G. uralensis* cultivation because of competition from other crops, whereas others may expand their cultivation because of favorable market prospects. This fluctuation in planting area reflects the complex relationship between the market environment and farmers’ decision-making and indicates a certain degree of vulnerability in the *G. uralensis* farming industry. In summary, the change in the *G. uralensis* distribution area in Dengkou County resulted from the combined action of natural conditions and human factors. In the future, in order to promote the sustainable development of *G. uralensis* plantations, it is necessary to continue to pay attention to and study the growth characteristics of *G. uralensis* and market demand and to seek reasonable planting strategies to achieve sustainable development.

## 5 Conclusions

As countries worldwide pay more attention to the development of the pharmaceutical industry, scientific management and sustainable development of medicinal plant resources, as the basis of the pharmaceutical industry, are crucial for ensuring the healthy development of the global pharmaceutical industry. The sustainable development and management of medicinal plant resources, such as *G. uralensis*, are crucial for the pharmaceutical, food, and cosmetic industries. This study employed Gaofen-1 satellite imagery and machine learning algorithms to monitor and extract the spatial and temporal distribution of *G. uralensis* in Dengkou County, providing valuable insights into sustainable cultivation practices. First, through a comparative analysis of the spectral characteristics of the main feature types in Dengkou County under different bands and months of the GF-1 WFV image, it was observed that the near-infrared band is significantly different from other bands in reflectance values and curve features and has the largest difference in the number of reflectance values with the blue band. Therefore, the difference between the blue and near-infrared bands was calculated to increase the spectral curves of the feature types, which is more conducive to the identification and extraction of *G. uralensis* and other feature types. The GUVI was constructed using a combination of the near-infrared and blue bands. The contribution rate of band importance, it is found that the addition of the contribution rate of each band in 12 months can significantly increase the difference between the blue and NIR bands and the other two bands. In other words, combining the January–December data to construct a classification model was more conducive to the identification of *G. uralensis*. Second, the long time series data of GUVI and common vegetation indices were used as the feature sets to construct eight identification models of *G. uralensis* using the RF algorithm. By comparing the classification accuracy of the RF algorithm for the eight models, it was observed that the OA and KC of the model constructed by the GUVI were second only to the one model constructed by the DVI. In the aspect of *G. uralensis* UA, the model constructed with EVI as the feature, the model constructed with GNDVI as the feature, and the model constructed with GCVI as the feature, and the best performance in *G. uralensis* PA. By comparing the classification accuracies of the RF classification model constructed with the GUVI feature set and the SVM classification model, we observed that the RF classification model constructed with the GUVI feature set had better efficiency. Then, in order to identify the distribution area of *G. uralensis* in historical remote sensing images, the spectral curves of *G. uralensis* in major feature types and different growth years were obtained under the GUVI feature set from January to December 2022. The spectral curve characteristics of *G. uralensis* in Dengkou County under the GUVI model differed from other feature types and the spectral curves of *G. uralensis* across different growth years. Finally, the training and validation sets were selected under the GUVI feature set for 2014–2021 according to the curve characteristics. The *G. uralensis* distribution areas for the last eight years were obtained. The distribution area of *G. uralensis* in 2014–2022 was gradually distributed in the middle of Dengkou County from the initial distribution in the northeastern part of Dengkou County, and is now distributed in the southwestern part of the county. Initially, the distribution area gradually changed from small and scattered to large and concentrated. The area of *G. uralensis* showed a trend of increasing first and then decreasing. This study demonstrates that the GUVI-based RF model is a powerful tool for monitoring and identifying the distribution of *G. uralensis*. Its adaptability and efficiency make it suitable for broader applications, including other medicinal plants and regions with similar ecological characteristics. The results of this study provide a foundation for improving the sustainability and productivity of *G. uralensis* cultivation. By enabling precise monitoring of planting areas, the GUVI-based model offers practical benefits for policymakers, resource managers, and farmers. However, regional customization of the model is necessary to account for variations in environmental and anthropogenic factors. Future research should focus on: firstly, expanding the model’s application to other regions and medicinal plants. Secondly, integrating advanced technologies, such as hyperspectral imagery and deep learning, to enhance classification accuracy. Thirdly, addressing limitations related to data quality and noise by incorporating additional datasets and preprocessing techniques.

## Data Availability

The raw data supporting the conclusions of this article will be made available by the authors, without undue reservation.
